# Androgen deficiency in hypopituitary women: its consequences and management

**DOI:** 10.1007/s11154-024-09873-1

**Published:** 2024-01-19

**Authors:** Daniela Esposito, Åsa Tivesten, Catharina Olivius, Oskar Ragnarsson, Gudmundur Johannsson

**Affiliations:** 1 Department of Internal Medicine and Clinical Nutrition, Institute of Medicine, Sahlgrenska Academy, University of Gothenburg, Gröna Stråket 8, Gothenburg, 41345 Sweden; 2 Department of Endocrinology, Sahlgrenska University Hospital, Gothenburg, Sweden; 3 Sahlgrenska Academy, Wallenberg Centre for Molecular and Translational Medicine, Institute of Medicine, University of Gothenburg, Gothenburg, Sweden; 4 Wallenberg Laboratory for Cardiovascular and Metabolic Research, Department of Molecular and Clinical Medicine, Institute of Medicine, Sahlgrenska Academy, University of Gothenburg, Gothenburg, Sweden; 5 Department of Medicine, Hospital of Halland, Kungsbacka, Sweden

**Keywords:** Hypopituitarism, Hypogonadotrophic hypogonadism, Androgen deficiency, Management

## Abstract

Women with hypopituitarism have various degrees of androgen deficiency, which is marked among those with combined hypogonadotrophic hypogonadism and secondary adrenal insufficiency. The consequences of androgen deficiency and the effects of androgen replacement therapy have not been fully elucidated. While an impact of androgen deficiency on outcomes such as bone mineral density, quality of life, and sexual function is plausible, the available evidence is limited. There is currently no consensus on the definition of androgen deficiency in women and it is still controversial whether androgen substitution should be used in women with hypopituitarism and coexisting androgen deficiency. Some studies suggest beneficial clinical effects of androgen replacement but data on long-term benefits and risk are not available. Transdermal testosterone replacement therapy in hypopituitary women has shown some positive effects on bone metabolism and body composition. Studies of treatment with oral dehydroepiandrosterone have yielded mixed results, with some studies suggesting improvements in quality of life and sexual function. Further research is required to elucidate the impact of androgen deficiency and its replacement treatment on long-term outcomes in women with hypopituitarism. The lack of transdermal androgens for replacement in this patient population and limited outcome data limit its use. A cautious and personalized treatment approach in the clinical management of androgen deficiency in women with hypopituitarism is recommended while awaiting more efficacy and safety data.

## Introduction

Hypopituitarism or anterior pituitary insufficiency includes various degrees of endocrine loss of function that may include growth hormone (GH), gonadotropins, thyrotropin-stimulating hormone (TSH), and adrenocorticotropic hormone (ACTH) production and secretion [1]. The prevalence of hypopituitarism is similar among men and women, and might be increasing due to the increased detection of hypothalamic-pituitary disorders with the increasing use of imaging of the head [2]. The management of hypopituitarism is often complex as many have complete loss of anterior pituitary function or panhypopituitarism [1]. These patients need replacement therapy with L-thyroxine, sex steroids, and glucocorticoids, and some also require GH replacement. Female patients receive estrogen-progestin replacement therapy during fertile age that is usually discontinued at the age of menopause. This recommendation is not based on any data from women with hypopituitarism but is based on guidelines addressing estrogen treatment for women in general. Women with hypopituitarism and combined hypogonadotrophic hypogonadism and secondary adrenal insufficiency have severe androgen deficiency [3]. Its impact on their health and outcome is not well known, and studies on the efficacy and safety of androgen replacement in women with hypopituitarism are scarce. The aim of this narrative review is to summarize available evidence on the consequences of androgen deficiency in women with hypopituitarism and its management, drawing insights from androgen deficiency in other groups of women and the effect of their treatment. The review also demonstrates the need for more research related to androgens in women in general and severe androgen deficiency in women in particular.

## Morbidity and mortality among women with hypopituitarism

Women with hypopituitarism have more marked morbidity and mortality than men [4, 5]. In 1990, it was shown that adults with hypopituitarism receiving replacement therapy according to standard treatment at that time (i.e. without GH replacement) had excess mortality [6]. The mortality was later shown to be more marked in women than in men and that morbidity such as diabetes mellitus, hypertension, and abdominal adiposity is more marked among women than men [7, 8]. The reason for this is not clear but can be related to several factors: women of fertile age may not receive adequate estrogen replacement, cortisol exposure may be higher in women than in men receiving glucocorticoid replacement therapy, and younger women may have more severe GH deficiency than men. One other possible reason is that women may have a longer diagnostic delay and are often underdiagnosed and undertreated as seen in other pituitary diseases as well as in diabetes and coronary heart disease [9–11]. Further, women with hypopituitarism and, in particular, those with secondary adrenal insufficiency have severe androgen deficiency that is rarely being replaced.

## Androgen levels in healthy adult women

In women, androgens are secreted from the adrenal glands and the ovaries, and are also produced in peripheral tissues by local conversion of prohormones.

The production of sex steroids by endocrine organs is regulated by the hypothalamus and pituitary gland. Specifically, the pituitary gland secretes gonadotropins and ACTH, which in turn control the secretion of both androgens and androgen prohormones from the adrenal glands (mainly regulated by ACTH) and the ovaries (mainly regulated by luteinizing hormone and follicle-stimulating hormone).

The major circulating androgens in women are the prohormones dehydroepiandrosterone sulfate (DHEA-S), dehydroepiandrosterone (DHEA), and androstenedione as well as the active androgens testosterone and dihydrotestosterone (DHT). DHEA-S and androstenedione are mainly of adrenal origin but are also produced by the ovaries [12–14]. Testosterone is produced by the ovaries and the adrenals as well as via peripheral conversion of prohormones, while DHT mainly is formed within target tissues [14, 15]. In younger women, circulating testosterone derives in approximately equal amounts from the ovaries (25%) and the adrenals (25%), and around 50% derives from peripheral conversion of androstenedione [16]. In the circulation, testosterone and other unconjugated androgens are bound to proteins, including sex hormone-binding globulin (SHBG) and albumin. This binding regulates their transport, distribution, metabolism, and biological activity. Consequently, any influence on binding proteins (such as the rise in SHBG mediated by oral estrogen) affects the concentration of active androgens, although a full understanding of the role of protein binding remains incomplete [17, 18].

Blood levels of both DHEA and DHEA-S in women peak between 20 and 30 years of age, and then decrease markedly with age: by the seventh decade of life, concentrations are about 30% of peak levels [19, 20]. There is a similar pattern for androstenedione [19, 20], whereas the age-associated decline in testosterone levels is less pronounced [20]. Testosterone, but not DHT, levels vary across the menstrual cycle in younger women but clinical menopause has no clear impact on androgen levels [21, 22].

**Fig. 1:**
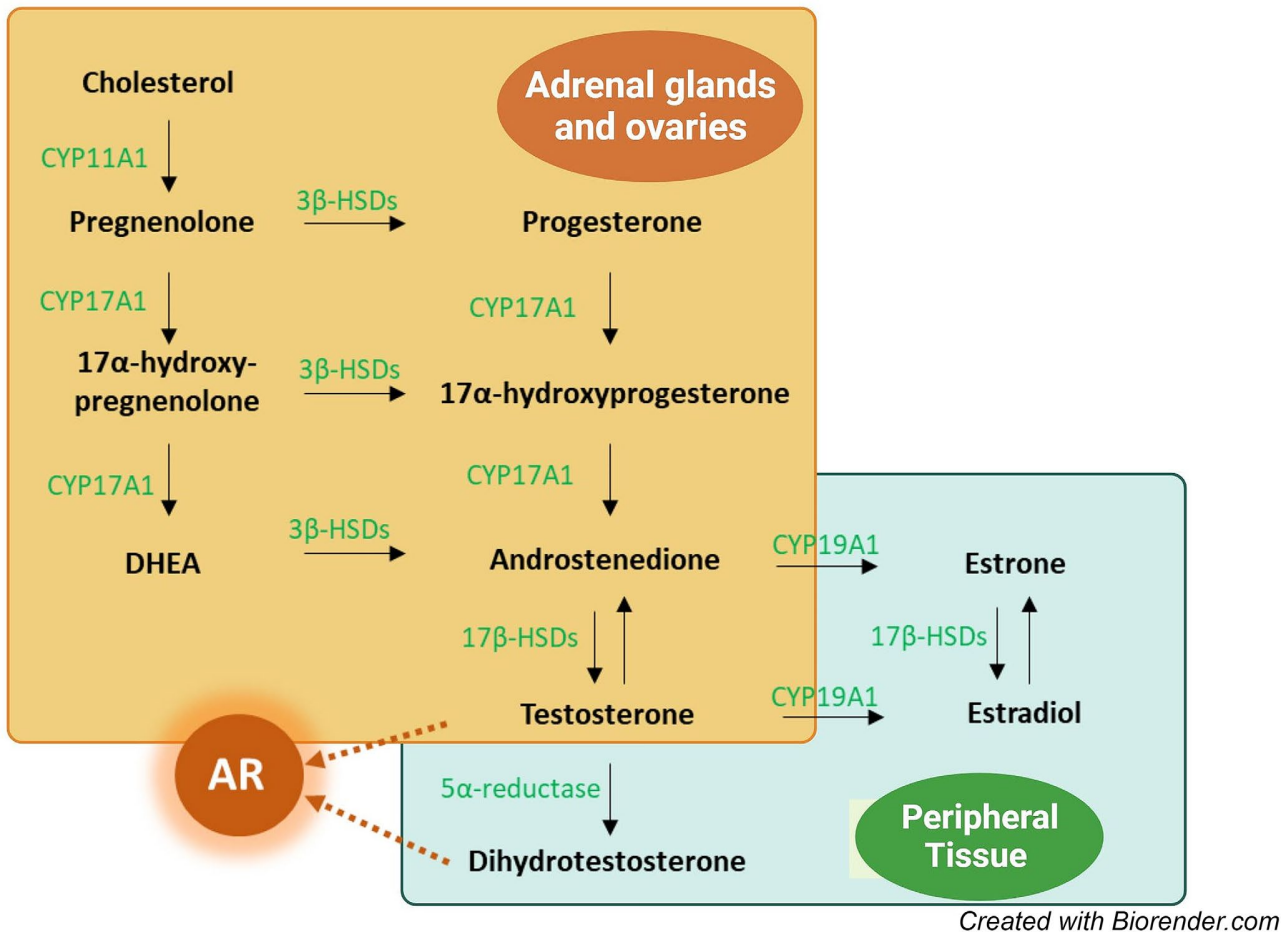
Simplified overview of androgen biosynthesis. Green text: most important sex steroid-metabolizing enzymes. AR: androgen receptor; CYP: cytochrome P450; DHEA: dehydroepiandrosterone; HSD: hydroxysteroid dehydrogenase

While classical endocrinology describes hormone production by endocrine organs that reach target tissues via the blood stream, the field of intracrinology describes the conversion of prohormones to active hormones within peripheral tissues. The enzymatic machinery to produce locally active androgens from androgen precursors (Fig. 1) is present in a large series of peripheral tissues and this mode of androgen production may be particularly important in women [23]. Peripheral tissues may also efficiently inactivate androgens, suggesting that circulating androgens may not reflect overall androgenic action [23]. Notably, the prohormones DHEA-S, DHEA, and androstenedione are much more abundant in female serum than testosterone and DHT [20]. Further, there is an increasing interest in 11-ketotestosterone, a potent androgen derived from 11-oxygenated adrenal androgen precursors, which is formed in peripheral tissues but also circulates at high levels in women independently of age [24].

Activation of the nuclear androgen receptor (AR), which is widely expressed in tissues, mediates a major part of the physiological actions of androgens. AR is stimulated either by testosterone or by its more potent metabolite DHT, which is produced within target cells by the 5-alpha reduction of testosterone (Fig. 1) [15]. 11-ketotestosterone is also a strong AR agonist [24], while DHEA and androstenedione have weak AR binding [15]. Other paths for the biological effects of androgens are via aromatization to estrogens (Fig. 1) or AR-independent androgenic signaling [15].

## Diagnosis of androgen deficiency in women with hypopituitarism

Women with diseases of the pituitary or hypothalamus, the adrenals, or the ovaries are at risk of developing androgen deficiency. However, progress in defining androgen deficiency, understanding its consequences, and optimizing its replacement have been hampered by the lack of sensitive and specific analytical methods for determining androgen levels. The performance of immunoassays that are commonly used in clinical routine is often insufficient for measurements of the low concentrations of sex steroids present in women and the use of mass spectrometry (MS)-based analytical methods are required [25–27]. To date, there is no established definition of androgen deficiency in women [22].

In hypopituitary women with both hypogonadotropic hypogonadism and ACTH deficiency, sex steroid production from both the adrenals and the ovaries is compromised. These women are likely to display a more severe androgen deficiency than women with isolated hypogonadotropic hypogonadism, isolated ACTH deficiency, or primary adrenal or ovarian failure. Indeed, a study using column chromatography-preceded radioimmunoassay reported that mean serum DHEA-S, androstenedione, and testosterone levels were markedly lower in women with both hypogonadotropic hypogonadism and ACTH deficiency compared to those with hypogonadotropic hypogonadism alone [28]. High-quality MS-based measurements of androgens in women with hypopituitarism may provide further important insights on their regulation but studies to generate these data have not yet been performed.

In women without pituitary disease, age- and cycle stage-specific reference intervals for androgens by liquid chromatography-tandem MS have been published [20–22, 29–31]. Notably, both estrogen [29, 32] and glucocorticoid [15] replacement may suppress endogenous androgen secretion, which should be taken into consideration when evaluating androgen levels in women with hypopituitarism.

## Clinical features related to androgen deficiency

The clinical features in patients with hypopituitarism are highly variable and depend on the underlying cause, which anterior pituitary hormones are affected, and the severity of the hormone deficiencies. The signs and symptoms of hypopituitarism are in most cases nonspecific and develop insidiously over weeks, months, or even years, especially when the underlying cause is a pituitary adenoma [33]. Furthermore, many symptoms (e.g. fatigue, malaise, and impaired general well-being) that women with hypopituitarism experience may be caused by ACTH-, TSH-, GH- and/or estrogen deficiencies as well as androgen deficiency. Thus, symptoms caused specifically by androgen deficiency may not be detected before other hormone deficiencies have been adequately diagnosed and treated.

**Fig. 2:**
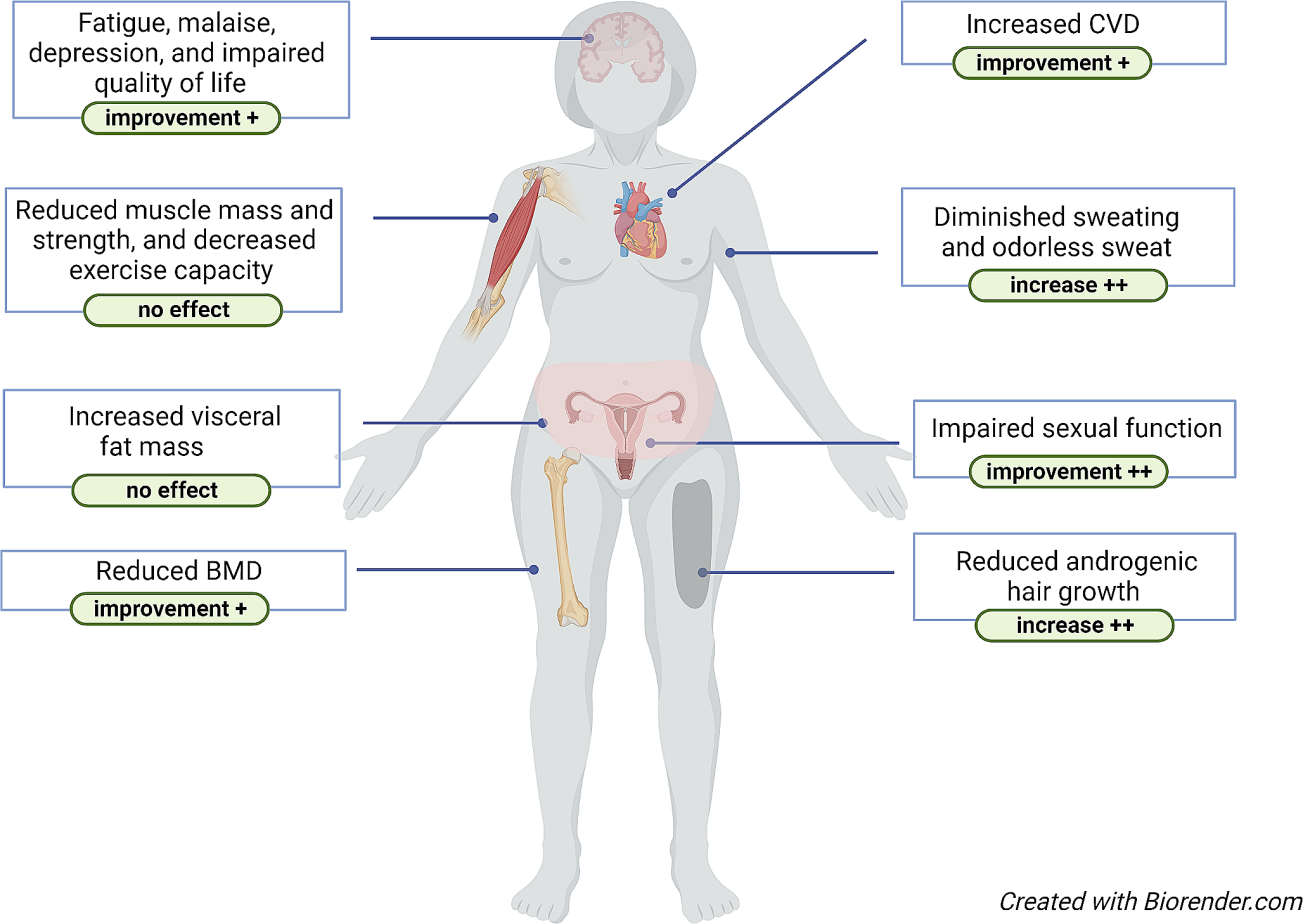
Clinical features in patients with androgen deficiency. Green boxes: effects of androgen replacement treatment. +: low evidence; ++: moderate evidence; BMD: bone mineral density; CVD: cardiovascular disease

Symptoms that are most strongly associated with androgen deficiency are related to reduced or absence of libido, i.e. reduced sexual arousal, fantasy, motivation, and enjoyment [34]. Other symptoms such as anxiety, depression, decreased alertness, decreased exercise capacity, and impaired general well-being and quality of life may also be associated with androgen deficiency (Fig. 2). Symptoms such as diminished sweating and odorless sweat are usually not noticed until treatment with androgen replacement has been started, when increased sweating, oily skin, and increased bodily odors may be experienced. On clinical examination a reduction of androgenic hair growth may be noticed as well as increased visceral fat mass, reduced muscle mass, and reduced muscle strength (Fig. 2).

## Associations between androgen levels and clinical outcomes in women

Potential associations between low androgen levels and cardiovascular disease (CVD), mortality, and fractures in women without overt endocrine disease may provide important clues on the potential role of androgen deficiency for the increased risk of these conditions in women with hypopituitarism.

Of note, cross-reactivity with, for example, inflammatory factors has been reported for immunoassays, which may result in false positive associations between androgen levels and disease in women [26]. Only a few studies have addressed associations between endogenous androgens measured by MS and CVD events and/or all-cause mortality in population-based cohorts of women. In one of those studies in older women [35], it was reported that lower levels of DHEA and testosterone were associated with higher risk of incident cardiovascular events but not all-cause mortality. In another study no association was observed between upper quartile levels of DHEA, androstenedione, or testosterone and incident CVD events among postmenopausal women [36]. In middle-aged women, higher DHT but not testosterone levels were associated with increased risk of all-cause mortality [37] whereas, in another study [38], no associations between androstenedione or testosterone and incident 10-year CVD or mortality risk among was found. Thus, studies of the association between high-quality measurements of androgen levels and CVD risk in women are few and report mixed results.

The main mechanism underlying CVD is atherosclerosis and a few recent MS-based studies lend support for a relation between low androgen levels and atherosclerosis in women. Among 2950 older women, lower testosterone and androstenedione levels were associated with a higher degree of coronary artery calcification, even after multivariate adjustment [39]. In women older than 55 years, lower DHEA and androstenedione were associated with increased carotid intima-media thickness after adjustment for traditional risk factors and DHEA was inversely associated with peripheral arterial disease [36]. In accordance with these clinical data, we have previously shown that AR deficiency in female mice results in increased atherosclerosis [40].

Importantly, the interpretation of association studies of androgen levels and outcomes in women is complicated by polycystic ovary syndrome (PCOS), affecting 5–18% of all women [41]. In PCOS, high levels of all androgens [42] coexist with an adverse cardiometabolic risk profile [41]. Some studies suggest that the relation between DHEA and testosterone with cardiovascular risk in women is U-shaped [43, 44], suggesting that both low and high androgen levels may be associated with CVD.

The effects of androgens on bone health are well known and low serum testosterone has been shown to be associated with low bone mineral density (BMD) in both middle-aged and older women [45–47]. The relative role of direct AR-mediated effects of testosterone on female bone and effects that depends on its aromatase-mediated conversion to estradiol remains unclear [48]. Genetically predicted higher serum DHEA-S was recently shown to increase lumbar BMD and decrease forearm fracture risk in women, strongly supporting a bone-protecting effect of endogenous DHEA-S in women [49]. Further, excess genetic risk for high testosterone levels in women with PCOS is associated with a higher BMD and reduced risk of fractures [50].

## Efficacy of androgen replacement in women with hypopituitarism

There is still controversial whether androgen replacement therapy should be practiced in women with hypopituitarism and coexisting androgen deficiency. Some studies suggest beneficial clinical effects of androgen replacement but data on long-term benefits and risk is not available [1]. Therefore, current guidelines from the US Endocrine Society do not recommend routine androgen treatment in women with androgen deficiency due to hypopituitarism [22].

## Testosterone replacement treatment

Hypoactive sexual desire disorder (HSDD) represents the only indication for evidence-based use of testosterone treatment in women, although there is scant data that androgen deficiency is responsible of this disorder [51, 52]. There is currently no established target level of testosterone in women to guide replacement therapy with testosterone and serum concentrations do not reliably predict treatment efficacy [53]. Of note, testosterone formulations intended for males are used in women since female-specific products are not approved or not accessible. Careful monitoring to ensure appropriate physiological dosing and minimize adverse effects is therefore needed. For optimal results and safety, it is suggested to use transdermal formulations of testosterone (i.e. patches or topical gels/creams), while oral products should be avoided due to documented unfavorable first-pass hepatic effects with adverse effects on sex hormone-binding globulin, thyroid-binding globulin, lipids, coagulation, and systemic inflammation [53].

A recent systematic review of blinded, randomized, controlled trials assessing safety and efficacy of testosterone therapy (for at least 12 weeks) in postmenopausal women showed a positive impact of this therapy on the frequency of satisfying sexual events, arousal, orgasm, pleasure, responsiveness, and self-image, while also reducing sexual concerns and distress [54]. Elevation in low-density lipoprotein cholesterol levels was observed with oral administration of testosterone, along with declines in total cholesterol, high-density lipoprotein cholesterol, and triglycerides [54]. However, these adverse effects on lipids were not observed when testosterone was administered as transdermal patches or creams. Additionally, testosterone treatment led to an overall increase in body weight. No impact of testosterone treatment was observed on body composition, musculoskeletal parameters, or cognitive functions, although available data from trials for these outcomes was limited [54]. This systematic review also showed that testosterone treatment was associated with mild androgenic effects on hair growth and acne [54]. Thus, despite beneficial clinical effects on sexual function of testosterone treatment in women, concerns about cardiometabolic safety have hindered its approval for treatment.

Data on the effect of testosterone treatment in women with androgen deficiency due to hypopituitarism is limited. The effect of testosterone treatment was assessed in a small study, involving 51 female patients with androgen deficiency due to hypopituitarism on oral estrogen replacement [55]. Transdermal testosterone replacement was administered at doses ranging from 150 to 300 µg/day over the course of 1 year, resulting in serum free testosterone concentrations within the normal range. Testosterone treatment led to an increase in mean bone mineral density at the hip and radius, but not in the spine. Other outcome measures included an increase in fat-free mass and thigh muscle area along with improvements in mood, sexual function, and overall quality of life. One-third of the women receiving testosterone reported developing acne, although no patients experienced hirsutism or other hyperandrogenic side effects [55]. In another study from the same group [56], testosterone replacement in hypopituitarism women was shown to have beneficial effects on cardiovascular risk factors and insulin resistance. Specifically, the study showed that the group of women on testosterone treatment for 12 months had lower fasting insulin and insulin-resistance homeostasis model of assessment in comparison to the placebo group. No effect of testosterone treatment was observed on high-sensitivity C-reactive protein, vascular cell adhesion molecule, leptin, lipoprotein (a), or apolipoprotein A1 [56].

## DHEA treatment

DHEA-S levels are reduced in women with primary and secondary adrenal insufficiency [22]. Whether treatment with DHEA is effective in improving fatigue, mood, or sexual function in women with adrenal insufficiency is controversial since only a few studies have investigated this issue.

Available data on the effects DHEA treatment in women with primary and/or secondary adrenal insufficiency from randomized, placebo-controlled studies is summarized in Table 1. Several, but not all, studies show beneficial effects on quality of life and sexual function [57, 59, 61]. On the other hand, some studies have shown an adverse effect on the lipid profile that could be related to the first-pass hepatic effects of the oral DHEA administration. Data concerning body composition, glucose metabolism, cardiovascular risk, and skeletal health is very limited. Furthermore, studies on the effects of long-term treatment with DHEA are still lacking. A meta-analysis, including 10 placebo-controlled trials assessing the effects of DHEA treatment in women with adrenal insufficiency showed that DHEA therapy led to a slight improvement in quality of life and a reduction in the rate of depression, but no significant effects on anxiety or sexual well-being were observed [71]. Based on this data, current guidelines do not recommend routine treatment with DHEA for women with low androgen levels due to hypopituitarism [22].

**Table 1:** Summary of randomized, placebo-controlled studies that have investigated the effects of DHEA replacement therapy in women with primary and/or secondary adrenal insufficiency. Only studies with ≥ 20 women are included

Author (year) [reference] Country	Patients	Treatment	Main findings
Arlt et al. (1999) [57] Arlt et al. (2000) [58] Callies et al. (2001) [59] Germany	24 women: 14 with PAI and 10 with SAI	DHEA 50 mg/day or placebo each for 4 mo	Improved general well-being, improved scores for anxiety and depression, and increased sexual interest and satisfaction with sex during DHEA treatment. Total and HDL-cholesterol decreased during treatment with DHEA. No effect on cognitive performance, carbohydrate metabolism, body composition, or exercise capacity.
Hunt et al. (2000) [60] UK	24 women (and 15 men) with PAI	DHEA 50 mg/day or placebo each for 3 mo	Increased self-esteem and improved overall well-being, mood, and fatigue during DHEA treatment. No effects on cognitive or sexual function, body composition, lipids, or bone mineral density.
Johannsson et al. (2002) [61] Sweden	38 women with SAI	DHEA 30 mg/day if age < 45 years and 20 mg/day if > 45 years or placebo for 6 mo	Improved alertness, stamina, and initiative during DHEA treatment. Sexual interest increased in women on DHEA 30 mg/day. Androgen effect on skin and hair growth seen in majority of DHEA-treated women. HDL-cholesterol and apolipoprotein A-1 decreased after DHEA.
Løvås et al. (2003) [62] Norway	39 women with PAI	DHEA 25 mg/day or placebo for 9 mo	No effects on subjective health status, sexuality, blood lipids, and markers of bone metabolism. 89% experienced side effects, in particular increased sweat odor and scalp itching.
Dhatariya et al. (2005) [63] USA	28 women with PAI	DHEA 50 mg/day or placebo each for 3 mo	DHEA increased insulin sensitivity and reduced total, LDL- and HDL-cholesterol and triglycerides.
Brooke et al. (2006) [64] UK	30 women (and 21 men) with hypopituitarism on GH replacement	DHEA 50 mg/day or placebo for 6 mo	DHEA replacement led to a modest improvement in psychological well-being.
Dhatariya et al. (2008) [65] USA	28 women with PAI	DHEA 50 mg/day or placebo each for 3 mo	DHEA had no effect on physical performance, body composition, protein metabolism, or muscle mitochondrial biogenesis.
Gurnell et al. (2008) [66] UK	62 women (and 44 men) with PAI	DHEA 50 mg/day or placebo for 12 mo	DHEA reversed ongoing loss of bone mineral density at the femoral neck and enhanced total body lean mass. No benefit on fatigue, cognitive function, or sexual function. No change in fat mass.
Binder et al. (2009) [67] Germany	23 young females (age 13–25 years) with SAI	DHEA 25 mg/day or placebo for 12 mo	Pubic hair growth increased (primary outcome) and psychological well-being improved during DHEA treatment.
Srinivasan et al. (2009) [68] USA	28 women with PAI	DHEA 50 mg/day or placebo each for 3 mo	Total and HDL-cholesterol decreased during DHEA treatment.
Rice et al. (2009) [69] UK	40 women: 20 with PAI and 20 with SAI	DHEA 50 mg/day or placebo each for 3 mo	Unchanged arterial stiffness and endothelial function during DHEA treatment.
Mandal et al. (2022) [70] India	28 women with SAI due to Sheehan’s syndrome	DHEA 50 mg/day or placebo each for 3 mo	Improved female sexual functioning index after DHEA treatment. Glycemic index, lipid profile, and liver enzymes unchanged on DHEA.

DHEA: dehydroepiandrosterone; GH: growth hormone; LDL: low-density lipoprotein; HDL: high-density lipoprotein; mo: month; PAI: primary adrenal insufficiency; RCT: randomized controlled trial; SAI: secondary adrenal insufficiency

## Conclusion

Women with hypopituitarism have various degrees of androgen deficiency, which is marked among those with combined hypogonadotrophic hypogonadism and secondary adrenal insufficiency. However, the available evidence of the importance of androgens in women and on the impact of androgen replacement therapy is limited. It is plausible, however, that androgen deficiency may adversely impact BMD, quality of life, libido, and sexual function in women. Compared to men, women with hypopituitarism have a more markedly increased disease burden with higher frequency of type 2 diabetes mellitus, CVD, and fractures. Whether this may be explained by unreplaced androgen deficiency in women is unclear. General recommendations for androgen replacement therapy cannot therefore be made but an individualized approach may be considered by offering androgen replacement for selected women with hypopituitarism with the target of normalizing serum testosterone concentration.

## Data Availability

No datasets were generated or analysed during the current study.

## Abbreviations

ACTH: Adrenocorticotropic hormone
AR: Androgen receptor
BMD: Bone mineral density
CVD: Cardiovascular disease
CYP: Cytochrome P450
DHEA: Dehydroepiandrosterone
DHEA-S: Dehydroepiandrosterone sulfate
DHT: Dihydrotestosterone
GH: Growth hormone
HDL: High-density lipoprotein
HSD: Hydroxysteroid dehydrogenase
HSDD: Hypoactive sexual desire disorder
LDL: Low-density lipoprotein
MS: Mass spectrometry
PCOS: Polycystic ovary syndrome
TSH: Thyrotropin-stimulating hormone

## References

[1] Fleseriu M, Hashim IA, Karavitaki N (2016). Hormonal replacement in hypopituitarism in adults: an endocrine Society Clinical Practice Guideline. J Clin Endocrinol Metab.

[2] Esposito D, Olsson DS, Ragnarsson O (2019). Non-functioning pituitary adenomas: indications for pituitary surgery and post-surgical management. Pituitary.

[3] Olivius C, Landin-Wilhelmsen K, Olsson DS (2018). Prevalence and treatment of central hypogonadism and hypoandrogenism in women with hypopituitarism. Pituitary.

[4] van Bunderen CC, Olsson DS (2023). Meta-analysis of mortality in adults with growth hormone deficiency: does growth hormone replacement therapy really improve mortality rates?. Best Pract Res Clin Endocrinol Metab.

[5] Pappachan JM, Raskauskiene D, Kutty VR (2015). Excess mortality associated with hypopituitarism in adults: a meta-analysis of observational studies. J Clin Endocrinol Metab.

[6] Rosén T, Bengtsson BA (1990). Premature mortality due to cardiovascular disease in hypopituitarism. Lancet.

[7] Sherlock M, Ayuk J, Tomlinson JW (2010). Mortality in patients with pituitary disease. Endocr Rev.

[8] Olsson DS, Bryngelsson IL, Ragnarsson O (2016). Higher incidence of morbidity in women than men with non-functioning pituitary adenoma: a Swedish nationwide study. Eur J Endocrinol.

[9] Shah T, Palaskas N, Ahmed A (2016). An update on gender disparities in coronary heart disease care. Curr Atheroscler Rep.

[10] Arnetz L, Ekberg NR, Alvarsson M (2014). Sex differences in type 2 diabetes: focus on disease course and outcomes. Diabetes Metab Syndr Obes.

[11] Esposito D, Ragnarsson O, Johannsson G (2020). Prolonged diagnostic delay in acromegaly is associated with increased morbidity and mortality. Eur J Endocrinol.

[12] Nunes E, Gallardo E, Morgado-Nunes S (2023). Steroid hormone levels in postmenopausal hysterectomised women with and without ovarian conservation: the continuous endocrine function of the ovaries. J Obstet Gynaecol.

[13] Wierman ME, Kiseljak-Vassiliades K (2022). Should dehydroepiandrosterone be administered to women?. J Clin Endocrinol Metab.

[14] van Winden LJ, Vermeulen RFM, van den Noort V (2022). Changes in sex steroids and relation with menopausal complaints in women undergoing risk-reducing salpingo-oophorectomy. J Endocr Soc.

[15] Alemany M (2022). The roles of androgens in humans: Biology, metabolic regulation and health. Int J Mol Sci.

[16] Shiffer L, Barnard L, Baranowski E (2019). Human steroid biosynthesis, metabolism and excretion are differentially reflected by serum and urine steroid metabolomes: a comprehensive review. J Steroid Biochem Mol Biol.

[17] Handelsman DJ (2017). Free testosterone: pumping up the tires or ending the free ride?. Endocr Rev.

[18] Goldman AL, Bhasin S, Wu FCW (2017). A reappraisal of testosterone’s binding in circulation: physiological and clinical implications. Endocr Rev.

[19] Labrie F, Luu-The V, Labrie C (2003). Endocrine and intracrine sources of androgens in women: inhibition of breast cancer and other roles of androgens and their precursor dehydroepiandrosterone. Endocr Rev.

[20] Mezzullo M, Gambineri A, Di Dalmazi G (2021). Steroid reference intervals in women: influence of menopause, age and metabolism. Eur J Endocrinol.

[21] Rothman MS, Carlson NE, Xu M (2011). Reexamination of testosterone, dihydrotestosterone, estradiol and estrone levels across the menstrual cycle and in postmenopausal women measured by liquid chromatography-tandem mass spectrometry. Steroids.

[22] Wierman ME, Arlt W, Basson R (2014). Androgen therapy in women: a reappraisal: an Endocrine Society clinical practice guideline. J Clin Endocrinol Metab.

[23] Labrie F, Martel C, Bélanger A (2017). Androgens in women are essentially made from DHEA in each peripheral tissue according to intracrinology. J Steroid Biochem Mol Biol.

[24] Storbeck KH, O’Reilly MW (2023). The clinical and biochemical significance of 11-oxygenated androgens in human health and disease. Eur J Endocrinol.

[25] Hsing AW, Stanczyk FZ, Bélanger A (2007). Reproducibility of serum sex steroid assays in men by RIA and mass spectrometry. Cancer Epidemiol Biomarkers Prev.

[26] Ohlsson C, Nilsson ME, Tivesten A (2013). Comparisons of immunoassay and mass spectrometry measurements of serum estradiol levels and their influence on clinical association studies in men. J Clin Endocrinol Metab.

[27] Handelsman DJ, Wartofsky L (2013). Requirement for mass spectrometry sex steroid assays in the Journal of Clinical Endocrinology and Metabolism. J Clin Endocrinol Metab.

[28] Miller KK, Sesmilo G, Schiller A (2001). Androgen deficiency in women with hypopituitarism. J Clin Endocrinol Metab.

[29] Haring R, Hannemann A, John U (2012). Age-specific reference ranges for serum testosterone and androstenedione concentrations in women measured by liquid chromatography-tandem mass spectrometry. J Clin Endocrinol Metab.

[30] Daan NM, Jaspers L, Koster MP (2015). Androgen levels in women with various forms of ovarian dysfunction: associations with cardiometabolic features. Hum Reprod.

[31] Fenske B, Kische H, Gross S (2015). Endogenous androgens and sex hormone-binding globulin in women and risk of metabolic syndrome and type 2 diabetes. J Clin Endocrinol Metab.

[32] Zimmerman Y, Eijkemans MJ, Coelingh Bennink HJ (2014). The effect of combined oral contraception on testosterone levels in healthy women: a systematic review and meta-analysis. Hum Reprod Update.

[33] Esposito D, Johannsson G, Ragnarsson O, Honegger J, Reincke M, Petersenn S (2021). Endocrinological diagnosis and replacement therapy for hypopituitarism. Pituitary tumors: a comprehensive and interdisciplinary approach.

[34] Zamponi V, Lardo P, Maggio R (2021). Female sexual dysfunction in primary adrenal insufficiency. J Clin Med.

[35] Islam RM, Bell RJ, Handelsman DJ (2022). Associations between blood sex steroid concentrations and risk of major adverse cardiovascular events in healthy older women in Australia: a prospective cohort substudy of the ASPREE trial. Lancet Healthy Longev.

[36] Meun C, Franco OH, Dhana K (2018). High androgens in postmenopausal women and the risk for atherosclerosis and cardiovascular disease: the Rotterdam Study. J Clin Endocrinol Metab.

[37] Schederecker F, Cecil A, Prehn C (2020). Sex hormone-binding globulin, androgens and mortality: the KORA-F4 cohort study. Endocr Connect.

[38] Schaffrath G, Kische H, Gross S (2015). Association of sex hormones with incident 10-year cardiovascular disease and mortality in women. Maturitas.

[39] Aribas E, Ahmadizar F, Mutlu U (2022). Sex steroids and markers of micro- and macrovascular damage among women and men from the general population. Eur J Prev Cardiol.

[40] Fagman JB, Wilhelmson AS, Motta BM (2015). The androgen receptor confers protection against diet-induced atherosclerosis, obesity, and dyslipidemia in female mice. FASEB J.

[41] Joham AE, Norman RJ, Stener-Victorin E (2022). Polycystic ovary syndrome. Lancet Diabetes Endocrinol.

[42] Stener-Victorin E, Holm G, Labrie F (2010). Are there any sensitive and specific sex steroid markers for polycystic ovary syndrome?. J Clin Endocrinol Metab.

[43] Laughlin GA, Goodell V, Barrett-Connor E (2010). Extremes of endogenous testosterone are associated with increased risk of incident coronary events in older women. J Clin Endocrinol Metab.

[44] Ohlsson C, Vandenput L, Tivesten A (2015). DHEA and mortality: what is the nature of the association?. J Steroid Biochem Mol Biol.

[45] Zhang H, Ma K, Li RM (2022). Association between testosterone levels and bone mineral density in females aged 40–60 years from NHANES 2011–2016. Sci Rep.

[46] Rariy CM, Ratcliffe SJ, Weinstein R (2011). Higher serum free testosterone concentration in older women is associated with greater bone mineral density, lean body mass, and total fat mass: the cardiovascular health study. J Clin Endocrinol Metab.

[47] Nunes E, Gallardo E, Morgado-Nunes S (2023). Steroid hormone levels and bone mineral density in women over 65 years of age. Sci Rep.

[48] Hammes SR, Levin ER (2019). Impact of estrogens in males and androgens in females. J Clin Invest.

[49] Quester J, Nethander M, Eriksson A (2022). Endogenous DHEAS is causally linked with lumbar spine bone mineral density and forearm fractures in women. J Clin Endocrinol Metab.

[50] Deshmukh H, Shah N, Papageorgiou M (2022). Genetic risk for the polycystic ovary syndrome, bone mineral density and fractures in women and men: a UK Biobank mendelian randomisation study. Bone.

[51] Davis SR, Baber R, Panay N (2019). Global consensus position statement on the use of testosterone therapy for women. J Clin Endocrinol Metab.

[52] Basson R (2021). Sexual dysfunctions in women: are androgens at fault?. Endocrinol Metab Clin North Am.

[53] Whitton K, Baber R (2023). Androgen-based therapies in women. Best Pract Res Clin Endocrinol Metab.

[54] Islam RM, Bell RJ, Green S (2019). Safety and efficacy of testosterone for women: a systematic review and meta-analysis of randomised controlled trial data. Lancet Diabetes Endocrinol.

[55] Miller KK, Biller BM, Beauregard C (2006). Effects of testosterone replacement in androgen-deficient women with hypopituitarism: a randomized, double-blind, placebo-controlled study. J Clin Endocrinol Metab.

[56] Miller KK, Biller BM, Schaub A (2007). Effects of testosterone therapy on cardiovascular risk markers in androgen-deficient women with hypopituitarism. J Clin Endocrinol Metab.

[57] Arlt W, Callies F, van Vlijmen JC (1999). Dehydroepiandrosterone replacement in women with adrenal insufficiency. N Engl J Med.

[58] Arlt W, Callies F, Allolio B (2000). DHEA replacement in women with adrenal insufficiency: Pharmacokinetics, bioconversion and clinical effects on well-being, sexuality and cognition. Endocr Res.

[59] Callies F, Fassnacht M, van Vlijmen JC (2001). Dehydroepiandrosterone replacement in women with adrenal insufficiency: effects on body composition, serum leptin, bone turnover, and exercise capacity. J Clin Endocrinol Metab.

[60] Hunt PJ, Gurnell EM, Huppert FA (2000). Improvement in mood and fatigue after dehydroepiandrosterone replacement in Addison’s disease in a randomized, double blind trial. J Clin Endocrinol Metab.

[61] Johannsson G, Burman P, Wirén L (2002). Low dose dehydroepiandrosterone affects behavior in hypopituitary androgen-deficient women: a placebo-controlled trial. J Clin Endocrinol Metab.

[62] Løvås K, Gebre-Medhin G, Trovik TS (2003). Replacement of dehydroepiandrosterone in adrenal failure: no benefit for subjective health status and sexuality in a 9-month, randomized, parallel group clinical trial. J Clin Endocrinol Metab.

[63] Dhatariya K, Bigelow ML, Nair KS (2005). Effect of dehydroepiandrosterone replacement on insulin sensitivity and lipids in hypoadrenal women. Diabetes.

[64] Brooke AM, Kalingag LA, Miraki-Moud F (2006). Dehydroepiandrosterone improves psychological well-being in male and female hypopituitary patients on maintenance growth hormone replacement. J Clin Endocrinol Metab.

[65] Dhatariya KK, Greenlund LJ, Bigelow ML et al. Dehydroepiandrosterone replacement therapy in hypoadrenal women: Protein anabolism and skeletal muscle function. Mayo Clin Proc. 2008;83(11):1218–25. 10.4065/83.11.1218. 10.4065/83.11.1218 PMC2753533 18990320

[66] Gurnell EM, Hunt PJ, Curran SE (2008). Long-term DHEA replacement in primary adrenal insufficiency: a randomized, controlled trial. J Clin Endocrinol Metab.

[67] Binder G, Weber S, Ehrismann M (2009). Effects of dehydroepiandrosterone therapy on pubic hair growth and psychological well-being in adolescent girls and young women with central adrenal insufficiency: a double-blind, randomized, placebo-controlled phase III trial. J Clin Endocrinol Metab.

[68] Srinivasan M, Irving BA, Dhatariya K (2009). Effect of dehydroepiandrosterone replacement on lipoprotein profile in hypoadrenal women. J Clin Endocrinol Metab.

[69] Rice SP, Agarwal N, Bolusani H (2009). Effects of dehydroepiandrosterone replacement on vascular function in primary and secondary adrenal insufficiency: a randomized crossover trial. J Clin Endocrinol Metab.

[70] Mandal S, Mukhopadhyay P, Ghosh S (2022). DHEA on sexual function in Sheehan syndrome: a randomized double-blind placebo-controlled crossover trial. J Clin Endocrinol Metab.

[71] Alkatib AA, Cosma M, Elamin MB (2009). A systematic review and meta-analysis of randomized placebo-controlled trials of DHEA treatment effects on quality of life in women with adrenal insufficiency. J Clin Endocrinol Metab.

